# New Insights on the Role of Sodium in the Physiological Regulation of Blood Pressure and Development of Hypertension

**DOI:** 10.3389/fcvm.2019.00136

**Published:** 2019-09-16

**Authors:** Erietta Polychronopoulou, Philippe Braconnier, Michel Burnier

**Affiliations:** Service of Nephrology and Hypertension, Department of Medicine, Centre Hospitalier Universitaire Vaudois, Lausanne, Switzerland

**Keywords:** skin, muscle, sweat, macrophages, immunity, microbiome

## Abstract

A precise maintenance of sodium and fluid balance is an essential step in the regulation of blood pressure and alterations of this balance may lead to the development of hypertension. In recent years, several new advances were made in our understanding of the interaction between sodium and blood pressure regulation. The first is the discovery made possible with by new technology, such as ^23^Na-MRI, that sodium can be stored non-osmotically in tissues including the skin and muscles particularly when subjects are on a high sodium diet or have a reduced renal capacity to excrete sodium. These observations prompted the refinement of the original model of regulation of sodium balance from a two-compartment model comprising the extracellular fluid within the intravascular and interstitial spaces to a three-compartment model that includes the intracellular space of some tissues, most prominently the skin. In this new model, the immune system plays a role, thereby supporting many previous studies indicating that the immune system is a crucial co-contributor to the maintenance of hypertension through pro-hypertensive effects in the kidney, vasculature, and brain. Lastly, there is now evidence that sodium can affect the gut microbiome, and induce pro-inflammatory and immune responses, which might contribute to the development of salt-sensitive hypertension.

## Introduction

Blood pressure (BP) may appear as a very simple physiological parameter defined as the product of cardiac output and peripheral arterial resistance. Yet, the regulation of BP is a highly complex, multi-facet interplay between renal, neural, cardiac, vascular, and endocrine factors under the influence of genetic and environmental factors (1). Thus, the precise mechanism whereby some individuals develop an elevated BP leading to hypertension remains undetermined in a majority of them. The Mosaic Theory of hypertension described by Page in 1960 (2), which included interactions among genetics, environment, adaptive, neural, mechanical, and hormonal perturbations (sympathetic nervous system, renin-angiotensin-aldosterone system) as the basis of hypertension, has been substantially modified in 2014 (1). It should probably be adapted further to include new players like the skin, the muscles, the immune system and the microbiome (3). Indeed, several important experimental and clinical studies have brought new insights into the possible role of these factors in the physiological regulation of BP. These new regulatory mechanisms may also begin to explain crucial involvement of the immune system in the development of salt-sensitive forms of hypertension for which there is ample evidence, but few postulated mechanisms (4, 5).

## Sodium and BP Regulation: From a 2- to a 3-compartment Model Including the Skin and Muscles

In 1972, Dahl reported the important correlation between dietary salt consumption and hypertension (6) and Guyton developed a complex model of BP regulation, in which the kidney is the key regulator maintaining the balance between sodium intake, extracellular volume and BP. He introduced the important concept of pressure natriuresis as the mechanism through which the kidney has the ability to preserve a normal BP through its functions to regulate volume homeostasis and sodium reabsorption (7, 8). His hypothesis consists essentially of a two-compartment model with the extracellular fluid volume within the intravascular space being in equilibrium with the interstitial space volume. Sodium being the major cation in the extracellular fluid, any change in urinary sodium excretion would lead to an increase in the intravascular fluid volume, thereby increasing BP and in some cases inducing hypertension.

The two-compartment model has been challenged in recent years due to two major factors. First, the observation that on a fixed sodium intake total-body Na+ content could exceed weight gain, suggesting that sodium accumulated without being osmotically active and that salt was stored in a third body compartment (9). The second important factor was the possibility of measuring tissue sodium content in muscles and skin using ^23^Na-magnetic resonance imaging (MRI) (10).

## The Non-osmotic Storage of Salt in Muscles and Skin

**Figure 1:**
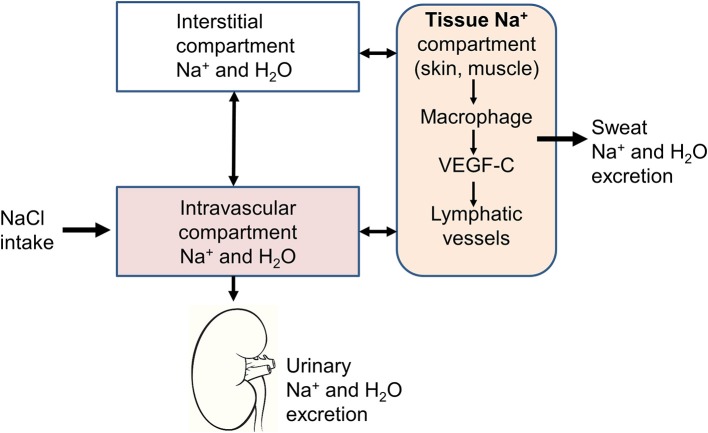
Schematic representation of the three-compartment model. In addition to the intravascular and interstitial compartments, sodium is stored in tissues, such as the skin or muscles. The sodium stored in this third compartment is not osmotically active and can be either mobilized to return to the intravascular compartment through lymphatic vessels or excreted through the sweat.

The traditional physiological concept placing the kidney in the very center of the regulation of extracellular volume and BP homeostasis, has been challenged by the group of Titze et al. after studying a group of astronauts simulating a long-term flight to Mars (9). They had the opportunity to expose this group of astronauts to different constant salt diets (6, 9, and 12 g/day) during 35 days and to perform simultaneously daily 24 h urine collections. To their great surprise, although salt intake was fixed, they noticed large variations in urinary sodium excretion. Changes in total-body Na^+^ exhibited rhythmic fluctuation within a day, which were not associated with parallel changes in body weight or extracellular water. However, the variations correlated positively with urinary aldosterone excretion and inversely to urinary cortisol. Toward the end of the observation period the total-body Na^+^ content exceeded the weight gain, suggesting that sodium had accumulated in another compartment without being osmotically active (Figure 1).

Skeletal muscle and skin are the body's major extracellular fluid compartments. Animal studies revealed that sodium is stored in the skin without concomitant water accumulation, bound to negatively charged glycosaminoglycan (GAG) (11–13). Skin GAG content can be directly measured by Western blot analysis and the Na^+^ concentration in skin can be determined by dry ashing and subsequent measurements of cations with atomic absorption spectrometry. Experimentally, the osmotically inactive skin Na^+^ can be mobilized by salt deprivation, which induces a reduction of the negatively charged skin glycosaminoglycan content (9). Furthermore, dietary salt loading is associated with an increased synthesis of GAG in the skin. These observations suggest that the storage of osmotically inactive Na in the skin is an active process. Skin sodium is stored directly under the keratinocyte layer in a microenvironment that is hypertonic to plasma suggesting sodium gradient formation in a kidney-like countercurrent system (14). In fact, in contrast to the lymph, which is isosmotic compared to the plasma, the skin is hyperosmotic and can control its own electrolyte microenvironment by creating a urea gradient from the epidermis to the dermis (15).

Interestingly, the sodium content in the interstitium seems to be regulated by the immune system through local modulations of the capillary lymphatic system in the skin (16). The skin phagocytes sense the hypertonic accumulation of sodium in the skin and this leads to an activation of the tonicity-responsive enhancer-binding protein (TONEBP, also known as NFAT5) and initiates the expression and secretion of VEGFC (vascular endothelial growth factor C). This latter has a double effect to increase the electrolyte clearance via cutaneous lymph vessels and to stimulate eNOS expression in blood vessels. Thereby immune cells exert a homeostatic and BP-regulatory control of interstitial electrolyte clearance via TONEBP and VEGFC and their respective impact on cutaneous lymphatic capillary function. Interestingly, mononuclear phagocyte system cell depletion or VEGF-C trapping blocks VEGF-C signaling and leads to sodium accumulation in the skin and elevated BP in response to high salt diet (17). Therefore, this new regulatory mechanism may contribute to the development of salt-sensitive forms of hypertension. Clinical studies have shown an age-dependent increase in skin tissue Na^+^ content that is associated with lower circulating VEGF-C levels, suggesting that VEGF-C enhances skin electrolyte clearance (18). Keratinocytes regulate skin perfusion by changing the balance between two hypoxia-inducible factor (HIF) transcription factor isoforms, HIF-1α and HIF-2α activity, and thereby regulate systemic arterial pressure by nitric oxide (NO) -dependent mechanisms (19, 20). In patients with essential hypertension, increased BP levels are associated with reduced NO levels in the skin secondary to a reduction of cutaneous HIF-1α and an increase of HIF-2α.

Elevated concentrations of sodium have also been documented in skeletal muscles of animals with experimental hypertension and in hypertensive patients (10, 21). As observed in the skin, the sodium concentration measured in muscles was higher than that measured in the plasma and could be mobilized with specific treatments increasing salt elimination, such as diuretics or dialysis.

This new concept of regulation of sodium balance and extracellular volumes not only through the kidney but also skin and muscles, might question the utility of 24 h urine collections to estimate salt intake. In fact, due the biologic variability of the urinary excretion of sodium, a 3 g difference in salt intake per day is detected correctly through a 24 h urine collection in only 50% in a stringently controlled environment (22). For this reason, single 24 h urine collections at intakes ranging from 6 to 12 g salt per day are probably not suitable to detect a 3 g difference in individual salt intake and repeated collections should be done to assess sodium intake more accurately.

## Information From New Methods to Measure Sodium in Tissue

The development of new technologies to measure sodium content in tissues has been an important adjunct to studies supporting the hypothesis of a 3-compartment model. Today sodium content in tissues can be visualized and quantified directly in skeletal muscles and skin through the development of ^23^Na-magnetic resonance imaging (MRI). By coupling ^23^Na-MRI with traditional ^1^H-MRI, it is possible to demonstrate that sodium accumulates in the skin and muscles without concomitant water accumulation (23). In a cohort of 56 healthy subjects and 57 patients with essential hypertension, ^23^Na-MRI measurements showed that patients with refractory hypertension had an increased tissue Na^+^ content compared with normotensive controls suggesting that sodium storage in the skin is associated with hypertension (23). In addition, ^23^Na-MRI studies have also revealed that the sodium content in the skin and muscles increases with age, an observation going along with the higher prevalence of hypertension in elderly subjects. Men appear to have a higher sodium content in the skin than women and women have higher muscle sodium than skin sodium (24). Interestingly, in case of primary hyperaldosteronism, the sodium content in skin and muscles is also elevated and is reduced by adrenalectomy or the prescription of an aldosterone antagonist (10). High muscle sodium concentrations have also been measured in patients with type 2 diabetes on maintenance dialysis (25). In these patients, skin sodium correlates with left ventricular hypertrophy and insulin resistance and can be reduced during a dialysis session (25, 26).

## Does Sweat Have a Role in the Regulation of Sodium Balance?

Sweat- the major product of the skin- may also be involved in the control of sodium balance in humans. The major function of sweat is the regulation of body temperature, but sweat glands are also able to secrete water and salt through several channels (27). Astonishingly, the sweat gland has some similarity with the convoluted tubules of the kidney as cells of the secretory coil of sweat glands contain ion channels, pumps and co-transporters, such as the Na^+^-K^+^-2Cl^−^ cotransporter (NKCC1), Na^+^-K^+^-ATPase, Na^+^-H^+^ exchanger (NHE1), aquaporine-5 (AQP5) (28). The sweat duct itself expresses epithelial sodium channel (ENaC), Na^+^-K^+^-ATPase and NHE1 participating in the reabsorption of ions. In particular, ENaC is expressed strongly in all epidermal layers and is located on the apical side of membranes in eccrine glands and ducts, reabsorbing Na^+^ ions (29). In a detailed sodium balance study performed by Heer et al. in healthy volunteers on salt intakes of about 3, 12, and 32 g/day, skin losses of sodium and chloride by sweat were reported to be negligible (mean sweat sodium excretion varied between 2.88 ± 0.35 and 4.92 ± 0.28 mmol/day) and were independent from salt intake (30). However, the low number of participants and the cumbersome method of sweat collection (volunteers wore an all-body cotton suit for 24 h) limited the interpretation of this study as only 3 subjects underwent sweat testing. Sports sciences provide the most recent data about sweat electrolytes and their excretion in humans (excluding cystic fibrosis). Thus, in a study of 157 marathon runners, 20% presented sweat salt losses equivalent to 3.5 ± 0.6 g of NaCl per liter of sweat which means that during a race time of 4 h and with a sweat rate of 1 L/h, salt losses can be as high as 14 g of NaCl (31).

In a cross-over design, we have recently assessed muscle sodium content by ^23^Na-MRI in 38 healthy normotensive volunteers after 5 days of high-sodium diet (HS) (6 g of salt added to their normal diet) and 5 days of a low-sodium diet (LS). In a sub-group of 18 participants we conducted quantitative pilocarpine iontophoretic sweat collections and measured the sodium concentration in sweat (32). Under HS conditions, urinary sodium excretion, muscle and sweat sodium concentrations all increased significantly. Sweat sodium concentrations correlated positively with salt intake as estimated by 24 h urine sodium excretion. Plasma aldosterone and plasma renin activity were negatively associated with both muscle and sweat sodium content. These results indicate that sweat sodium excretion are significantly higher on a high salt intake in healthy subjects and suggest that sweat may also play a role in regulating sodium balance in humans. These findings extend those from studies of the expression of the mineralocorticoid receptor (MR) and 11β-hydroxysteroid dehydrogenase in sweat gland epithelium, as well as the relationship between MR expression, salt intake and aldosterone levels (33–37).

## Salt and the Immune System in Hypertension

**Figure 2:**
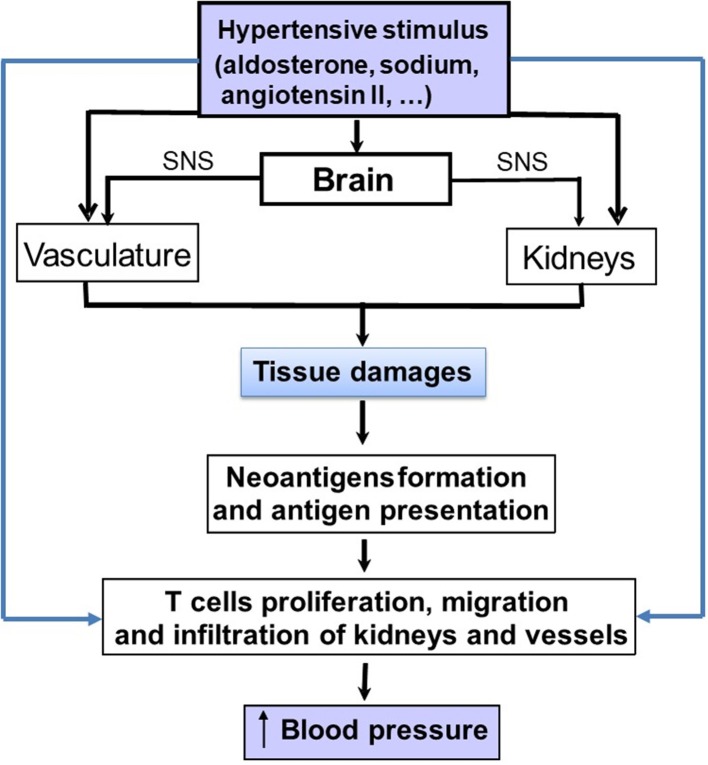
Schematic representation on how pro-hypertensive stimuli, such as aldosterone, angiotensin II or sodium can stimulate the immune system either directly or indirectly to increase blood pressure (BP). The indirect pathway involve the development of tissue lesions in the kidneys and vasculature. Damaged cells from these tissues generate cell particles acting as neo-antigens. These latter may induce an immune response with an activation of lymphocytes and the production of cytokines that will increase blood pressure.

The three-compartment model described above involved the immune system through immune cells including macrophages as important components leading to sodium storage or release from tissues where it has accumulated. Of course, this model does not exclude the central role of the kidney but add another regulatory system in the model, in which the immune system is involved. Today, most recent hypotheses on the pathophysiology of hypertension consistently include the immune system as a crucial co-contributor to the development of hypertension through pro-hypertensive effects in the kidney, vasculature, and brain (38–40) (Figure 2). Actually, the first time that the immune system was implicated in the process of hypertension generation was in 1954 by RH Heptinstall (41), who reported data on renal biopsies of hypertensive patients who showed early and scattered arteriolar hyalinization and intimal thickening of some small arteries. He also reported an accumulation of immune cells in kidney biopsies of hypertensive patient (41). The immune system is divided in two functional compartments: the innate immune system, which reacts rapidly, but is rather non-specific, with responses to a wide array of pathogens, and the adaptive immune system, which initiates slower but develops antigen-specific responses. Both components have been implicated as potential contributors to the development of various forms of hypertension, but mainly salt-sensitive hypertension (38, 42–45).

In experimental models, salt-sensitive hypertension is associated with increased renal expression of pro-inflammatory molecules including cytokines, chemokines and adhesion molecules (46–48). In addition, the inflammasome appears to contribute to the development of hypertension in renin-dependent and independent hypertension (49, 50) under the effect of salt, angiotensin II but also the sympathetic nervous system and endothelin (51). Four inflammasomes have been identified so far and the NLR family, pyrin domain-containing 3 (Nlrp3) inflammasome has been the most fully characterized. It forms a multi-protein complex with apoptosis-associated speck-like protein containing a caspase recruitment domain (Asc) and the protease Caspase-1, which activates the cytokines pro-interleukin-1β (IL-1β) and pro-IL-18. Conversely, pharmacological inhibition of inflammasome has been shown to lower BP in salt-sensitive hypertension (52, 53).

Several studies have also shown that angiotensin II has pro-inflammatory effects and can increase macrophage infiltration in the renal interstitium leading to a sustained elevation of BP, interstitial fibrosis, and preglomerular hypertrophy (53). Angiotensin II stimulates redoxi-sensitive-signaling cascades leading to mitogen activated protein kinase activation, activation of the p38 mitogen, an activated protein kinase, and increased oxidative stress (54, 55). This cascade stimulates the inflammatory mediators NFkB and activator protein-1 and further leads to production of chemokines involved in macrophage recruitment and prothrombotic agents, such as plasminogen activator inhibitor-1 and adhesion molecules (41, 42). In this context, T lymphocytes may have an important role to mediate the angiotensin II-induced hypertension (43). Thus, it has been postulated that the angiotensin II-induced organ damages generate neo-antigens from damaged cells leading to an immune reaction within the renal tissue and to the production of pro-inflammatory cytokines, such as TNF-α and IL-1β from infiltrating mononuclear cells. These cytokines may participate in the maintenance of hypertension and salt sensitivity through their effects on renal sodium handling (55). However, angiotensin II has also major effects on the vasculature. Thus, activation of AT_1_ receptors can cause an hemodynamic injury (52) that leads to further recruitment of monocytes into key effector tissues in hypertension, including the heart, the vascular bed and the kidney (48, 53, 54). Similarly, activation of the MR on cardiac and vascular cells, as well as immune cells, also increases immune cell-mediated effects, which in excess produce hypertension and deleterious cardiovascular and renal remodeling (56–58). Figure 2 summarizes the direct and indirect mechanisms whereby sodium, aldosterone and angiotensin II can activate the immune system in tissues.

Conversely, some studies showed that immune suppression lowered BP in rats with renal infarction and could disrupt the evolution of salt-sensitive hypertension (47, 48). Thus, the group of Rodriguez showed that during experimental hypertension, T cells infiltrate the kidney leading to the disruption of the nephron's capacity to excrete sodium and water and results in the elevation of BP (36). Inhibition of infiltration of T cells, using a lymphocyte-specific inhibitor (mycophenolate mofetil) decreases the renal infiltration of T cells, and improves BP and decreases kidney damage (36). In addition, the transfer of lymph node cells from hypertensive to normotensive rats induces hypertension in the recipients, and angiotensin II infusion causes vascular inflammation (44–46).

The three most important cytokines that play a crucial role are IL-17α, produced from Th17 cells, IFNγ and TNFα. IL-17-α or IFN deficiency can limit expression of sodium transporters in the proximal tubule, an effect which can facilitate the excretion of saline load (49). Experiments in Dahl salt-sensitive rats and a subset of hypertensive humans showed increased BP, albuminuria, and infiltration of macrophages and T cells in the kidneys in response to increased dietary sodium (49). Moreover, pro-inflammatory macrophage cytokines, TNF-α and IL-1β, both independently influence renal sodium handling in response to activation of the renin-angiotensin system (59). Experiments with TNF-deficient animals have shown that TNF-α potentiates renal sodium reabsorption in the kidney's thick ascending limb via nitric oxide synthase 3 (NOS3) suppression (51). According to Rucker and his colleagues (55) IL-1 receptor activation decreases the number of NO-expressing macrophages in the kidney and as consequence reduces inhibition of the NKCC2 sodium transporter by NO, thus favoring renal salt retention (59).

## Is Salt the Triggering Factor for the Pro-inflammatory Cascade?

One hypothesis linking sodium to the inflammatory pathway is that high salt leads to CD4^+^T cell proliferation and produces IL-17 related cytokines (59). These latter induce the secretion of IL-23, IL-6 and IL1β, and lead to IL17 production from T cells (59). This could result in renal and vascular inflammation, an impaired renal function, a shift of the pressure/natriuresis relationship and the development of hypertension (59). Thus, Zhang et al. showed that mouse and human macrophages cultured in a high salt environment produced more inflammatory and less anti-inflammatory cytokines than those cultured in normal salt (59). Besides, macrophages stimulated with IL-4 and IL-13 become less anti-inflammatory in the presence of a high-salt environment. *In vitro*, high salt has also been reported to alter protein phosphorylation, an effect, which could affect several important cellular functions and maybe inflammatory pathways (60). Interestingly, in a *post-hoc* analysis of the subjects having participated in the MARS project discussed previously, Yi et al. observed an increase in inflammatory cytokines (IL-6 and IL-23) and a decrease in the anti-inflammatory cytokine IL-10 in the plasma of subjects when on the highest salt intake when compared to the lower salt period (61). This observation suggests that in healthy humans a high-salt diet has a potential to induce an excessive immune response. Therefore, sodium intake itself could be one of the important triggering factor leading to inflammation in hypertension.

## Salt, Hypertension, Immunity, and the Gut Microbiome

**Figure 3:**
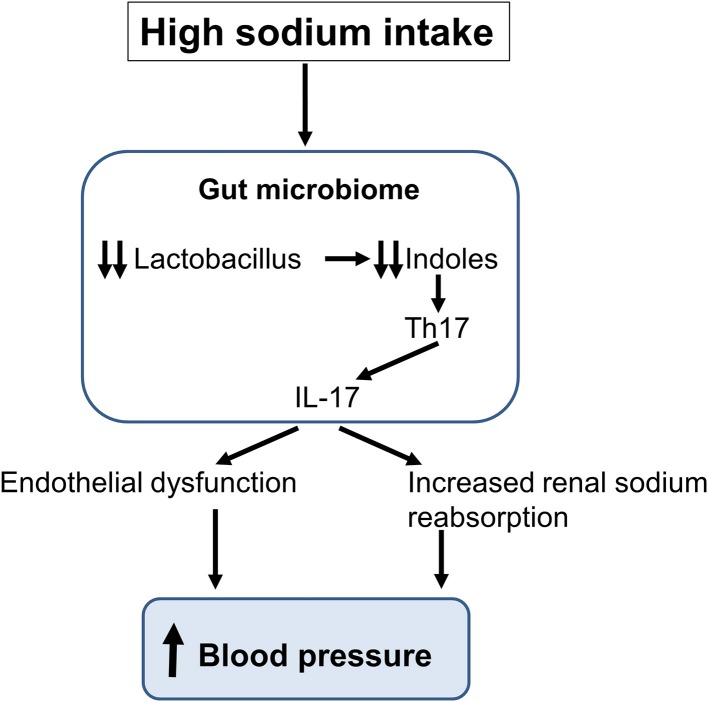
Schematic representation of the impact of a high sodium intake on the gut microbiome. The sodium-induced changes in gut microbiota lead to the production of interleukin-17 (IL-17) inducing an endothelial dysfunction and an increase in renal sodium reabsorption thereby increasing blood pressure.

In the last decade, the gut microbiota has been associated with the development of several diseases including cardio-metabolic diseases and it has been the subject of an intensive research (62, 63). Considering the impact of a high-salt intake on pro-inflammatory immune cells and the development of hypertension, it appeared logical to investigate the role of salt intake on the composition of the gut microbiota and the possible implication of this latter in the pathogenesis of hypertension. Recently, Wilck et al. (64) described a novel interaction between a high-salt intake and T cell phenotype which is mediated by changes in the composition of the gut microbiome with a depletion of Lactobacillus species and reduced generation of bacterial indoles (Figure 3) (65). Wyatt and Crowley (65) have assessed the role of Lactobacillus treatment on the development of salt-sensitive hypertension in mice. In these studies, mice on a high salt diet had an elevated BP, but this latter could be reduced with a concomitant treatment with Lactobacillus. When analyzing the T lymphocyte population in intestinal and splenic tissue, they found an increased frequency of Th17 lymphocytes in mice on a high-salt diet. Treatment with Lactobacillus enabled to reduce the number of Th17 lymphocytes in these tissues in animals on a high salt intake. Thus, a diet rich in sodium appears to affect intestinal microbiota, increasing intestinal Th17 cells. Together, these studies showed that modification of the gut microbiome by the excessive consumption of sodium increases the systemic inflammatory milieu (66). Moreover, analyzes of the gut microbiota in animals and humans with hypertension show similar modifications (65, 67–69).

## Conclusions

Several new aspects of the role of sodium in the regulation of sodium balance and in the development of hypertension have been revealed in the last 10 years as summarized in Table 1. There is now evidence that sodium contributes to the pathogenesis of hypertension through an effect on the immune system. Sodium modulates the immune cell function and a high salt micro-environment in tissues can cause local inflammation, tissue damages and in some cases hypertension. Sodium is stored in a non-osmotically active manner in the skin and muscles and may be excreted through the sweat in response to a high salt diet, a newly described mechanism involving tissue macrophages. This storage may actually protect from an excessive increase in BP, excluding sodium from the intravascular space.

**Table 1:** Major experimental and clinical observations having modified our understanding of the regulation of sodium balance and the role of sodium in the genesis of some forms of hypertension.

**Observations**		**References**
1.	Despite a fixed salt intake, there are large variations in 24 h urinary sodium excretion with rhythmic fluctuations in total-body Na^+^ within a day	(9)
2.	Sodium is stored non-osmotically in tissues like skin and muscles	(12, 13)
3.	Sodium content in skin and muscles can be measured by ^23^Na-magnetic resonance in animals and humans	(10, 22, 23)
4.	Skin and muscle sodium are elevated in elderly, in hypertensive patients and in patients with type 2 diabetes on dialysis. Diuretics, adrenalectomy and dialysis reduce tissue sodium.	(10, 23–25)
5.	Sweat participates in the regulation of sodium excretion	(32)
6.	The immune system participates in the development and maintenance of salt-sensitive forms of hypertension	(38, 40, 45, 47, 49, 55)
7.	A high sodium intake modifies the intestinal microbiota and may increase blood pressure via T-helper lymphocytes 17 and interleukin 17.	(64–68)

While small changes in plasma concentration of sodium are unlikely to induce inflammation, the hypothesis that larger and/or chronic increases in sodium trigger an inflammatory response is gaining more ground. However, as of today, one does not know precisely in which other tissues, besides skin and muscles, a high-salt environment may activate immune cells. Wiig et al. reported slight elevations in sodium concentrations in lymph capillaries in hypertensive rats (16). Another possible mechanism whereby salt would stimulate the immune system is that circulating antigen-presenting cells may be activated by high concentrations of sodium in peripheral tissues before turning into lymphoid tissues and activating T cells (70).

Further understanding of the exact mechanisms whereby sodium interacts with the immune system and gut microbiota might offer new opportunities for therapeutic approaches of hypertension with unexplored targets. A global immunosuppression of T lymphocytes may be excessive and associated with too many side effects and hence would not be appropriate to treat an asymptomatic disease, such as hypertension. Yet, specifically targeting key components regulating the T cell's contribution to BP regulation may still be an option, provided the therapy is safe and well-tolerated. Sustained modifications of the gut microbiota might represent another therapeutic approach that needs to be explored. However, today, reducing daily salt consumption to 5–6 g per day remains the easiest and most cost-effective way to limit the impact of sodium on blood pressure and to prevent the cardiovascular complications of hypertension.

## Author Contributions

MB, EP, and PB have written the paper. MB has done the figures.

### Conflict of Interest Statement

The authors declare that the research was conducted in the absence of any commercial or financial relationships that could be construed as a potential conflict of interest.
